# Unveiling the regulatory mechanism of poly-γ-glutamic acid on soil characteristics under drought stress through integrated metagenomics and metabolomics analysis

**DOI:** 10.3389/fmicb.2024.1387223

**Published:** 2024-05-01

**Authors:** Li Hong, Li Wei, Ge Fanglan, Li Jiao, Tu Shiheng, Yang Hong, Ren Yao, Gong Xinyue, Yao Can

**Affiliations:** ^1^Key Laboratory of the Evaluation and Monitoring of Southwest Land Resources (Ministry of Education), Sichuan Normal University, Chengdu, China; ^2^College of Life Sciences, Sichuan Normal University, Chengdu, China

**Keywords:** poly-γ-glutamic acid, soil characteristics, metagenome analysis, metabonomic analysis, drought stress

## Abstract

It is of utmost importance to understand the characteristics and regulatory mechanisms of soil in order to optimize soil management and enhance crop yield. Poly-γ-glutamic acid (γ-PGA), a stress-resistant amino acid polymer, plays a crucial role in plant drought stress resistance. However, little is known about the effects of γ-PGA on soil characteristics during drought treatments. In this study, the effects of different forms of γ-PGA on soil texture and basic physical and chemical properties under short-term drought conditions were investigated. Furthermore, the impact of γ-PGA on the microbial community and metabolic function of maize was analyzed. Under drought conditions, the introduction of γ-PGA into the soil resulted in notable improvements in the mechanical composition ratio and infiltration capacity of the soil. Concurrently, this led to a reduction in soil bulk density and improved soil organic matter content and fertility. Additionally, metagenomic analysis revealed that under drought conditions, the incorporation of γ-PGA into the soil enhanced the soil microbiota structure. This shift led to the predominance of bacteria that are crucial for carbon, nitrogen, and phosphorus cycles in the soil. Metabolomics analysis revealed that under drought treatment, γ-PGA affected soil metabolic patterns, with a particular focus on alterations in amino acid and vitamin metabolism pathways. Correlation analysis between the soil metagenome and metabolites showed that microorganisms played a significant role in metabolite accumulation. These results demonstrated that γ-PGA could improve soil characteristics under drought conditions and play an important role in soil microorganisms and microbial metabolism, providing further insights into the changes in soil characteristics under drought conditions.

## 1 Introduction

Drought is a major abiotic stressor that contributes to reduced crop yields (Zhang et al., 2022). In light of projected future climate conditions, it is anticipated that droughts that exceed historical ranges will become more frequent (Ault, 2020), posing significant challenges to agriculture (Mansoor et al., 2022). Soil texture has a direct effect on crop growth and yield (Qiao et al., 2022). Therefore, improving soil texture under drought conditions is essential to address impending drought challenges.

Poly-γ-glutamic acid (γ-PGA) is a non-toxic, water-soluble, biodegradable, and environmentally friendly biopolymer (Luo et al., 2016) that has been widely utilized in various fields including food, cosmetics, and medical care (Stephen Inbaraj et al., 2006; Hu et al., 2023). Furthermore, its potential for agricultural application has also been recognized. The γ-PGA can significantly increase the dry weight of roots and buds and the root-shoot ratio of cucumber seedlings (Wang et al., 2008), accelerate nitrogen metabolism in plants through the Ca2þ/CaM signaling pathway, and improve the growth of Chinese cabbage (Xu et al., 2014). In recent years, numerous reports have indicated that γ-PGA acts as a regulator of plant growth and stress resistance. Yin et al. (2018) reported that γ-PGA fermentation broth can significantly increase the biomass of maize seedlings and enhance their drought tolerance. Additionally, Xu et al. (2020) observed that under drought stress, rape seedlings treated with γ-PGA promoted abscisic acid accumulation, inducing the influence of tolerance system elements on drought stress. Moreover, γ-PGA can alleviate the stress caused by cold (Xu et al., 2017), heat (Quan et al., 2022), salt (Guo et al., 2017), metals (Wang et al., 2020), and stress defense, thereby enhancing the stress resilience of plants.

Soil, as the foundation of agricultural production and the carrier of its life system, plays a vital role in plant growth and development. Thus, the influence of γ-PGA on the soil has gained increasing attention. Liang et al. (2019) reported that adding γ-PGA to desertification-poor soil improved cotton growth and development, nitrogen and phosphorus absorption and efficiency, and water and fertilizer productivity. Chen et al. (2021) reported that treating saline soil with γ-PGA significantly reduced salinity and helped to retain more total nitrogen on the soil surface. Guo et al. (2021) demonstrated that γ-PGA and γ-PGA hydrogels can reduce the infiltration of stagnant water in sandy loam, thereby reducing the deep infiltration loss of soil moisture. In addition, Ma et al. (2022) suggested that γ-PGA promoted plant growth and bacterial accumulation in the rhizosphere soil. Although the effects of γ-PGA on soil properties have been widely studied, the impact of drought treatment on soil mechanical composition remains unexplored.

Soil non-targeted metabolomics can be used to characterize the differential activity of microbial communities (Abram, 2015) and reflect the interaction between the microbial genome and the environment (Tang, 2011), providing a novel approach for assessing soil health. For example, alterations in soil carbohydrate metabolite abundance are influenced by factors such as soil organic matter content and microbial activity (Duan et al., 2023). Therefore, soil metabolomics is valuable for assessing the impact on soil quality and microbial function (Withers et al., 2020). However, there is currently no literature documenting the composition and alterations in soil metabolites resulting from γ-PGA application.

This study examined the effects of γ-PGA application on soil structure and physical and chemical properties under drought conditions and conducted a comprehensive analysis of the soil metagenome and non-targeted metabolomics. These findings revealed the regulatory effect of γ-PGA on soil characteristics under drought conditions, with the aim of providing a scientific basis for sustainable dryland agriculture development.

## 2 Materials and methods

### 2.1 Experimental materials and pot experiment design

The corn variety examined was “Baitiannuoyujing,” and the soil samples were obtained from the farm of Sichuan Normal University, where Chinese Cabbage (*Brassica rapa L*. ssp. pekinensis) planted previously. Department of Agriculture (USDA) classification system, the soil type was determined to be silty soil, comprising 7.21% clay, 80.93% silt, and 11.82% sand. Soil was collected from a depth of 0–20 cm in March 2022. The samples were allowed to air dry naturally, and then ground and sifted through a 2 mm mesh screen. Based on the U.S.

The molecular weight of γ-PGA purified from *Bacillus subtilis* SCP010-1 fermentation broth in our laboratory (Chang et al., 2022) was approximately 1.1 million Da. To prepare the γ-PGA hydrogel, γ-PGA was dissolved in distilled water at a ratio of 10% (w/v), stirred evenly, and mixed with the cross-linking agent PEGDE (7: 500 = v/v). The mixture was then cross-linked for 6 h in a 60°C water bath and ground into particles with size of 1–2 mm. Biochar was purchased from Changge Catalysis Technology Co., Ltd. (Zhengzhou). At the 30th hour of the 40-h fermentation process to produce γ-PGA, sterilized biochar (biochar: culture medium = 1:5) was introduced. After fermentation, the resulting product was oven-dried at 90°C, ground into a powder.

The pot experiment was conducted in a greenhouse at Sichuan Normal University between April and May 2022. Prior to sowing, each material such as biochar, γ-PGA, γ-PGA hydrogel, fermentation broth containing γ-PGA, fertilizer (N-P2O5-K2O = 15:15:15) were uniformly mixed with soil by mass ratios of 0.2%, then were filled in plastic pots (10 cm × 9 cm × 14 cm), respectively. There were five treatments in the experiment: control (CK), including fertilizer; treatment 1 (T1), biochar plus fertilizer; treatment 2 (T2), γ-PGA hydrogel plus fertilizer; treatment 3 (T3), fermentation broth containing γ-PGA plus fertilizer; and treatment 4 (T4), biochar-adsorbing γ-PGA plus fertilizer, with 10 repetitions for each treatment. During the planting period, the temperature ranged from 18°C to 32°C, and the relative humidity ranged from 20 to 67%. Corn seeds were soaked for 12 h and then leached. Five seeds were planted in each pot and watered. At the one-leaf stage, two seedlings with the same growth status were left in each pot. At the stage of three leaves and one heart, seedlings were subjected to drought stress by withholding water. Soil moisture content was calculated daily using the weighing method. When it reached the level of severe drought (soil moisture content of 30–40%), the drought was terminated, and the seedlings were rewatered.

### 2.2 Sampling of rhizosphere soil samples

After shaking off the loosely bound soil, the tightly bound soil was collected from the rhizosphere of two plants and a sample was formed for subsequent analysis. Three biological replicates were randomly selected from each treatment for subsequent analysis. The soil samples were divided into two portions: one was placed in a refrigerator at −80°C for metagenomic and soil metabolomics analysis, while the other portion was air-dried under natural conditions and sifted through 20-mesh and 100-mesh screens to determine basic soil properties.

### 2.3 Determination of soil characteristics

A Malvin particle size analyzer (Malvern Mastersizer 3000, UK) was utilized to determine the mechanical composition of the soil in accordance with Equation (Liu et al., 2013) to calculate the classification dimension of the soil particle volume. The vertical infiltration experiment was conducted using a one-dimensional soil column (Guo et al., 2021) comprising a soil column (30 cm in height and 5 cm in inner diameter), a Mars bottle (50 cm in height and 5 cm in inner diameter), a soil loading height of 24 cm, and a constant water head of 2 cm. Additional soil properties were determined following the methods outlined in the Soil Agrochemical Analysis section (Lu, 1999). After air-drying and sieving through a 2 mm sieve, the soil bulk density was assessed using the ring knife method. Conductivity was measured using a conductivity meter. The soil organic matter content was determined using the potassium dichromate-external heating method. Urease activity was assessed using indophenol blue colorimetry, whereas soil sucrase activity was measured using 3-5-dinitrosalicylic acid colorimetry. Alkaline phosphatase activity was determined using a kit (Solarbio, China).

### 2.4 Metagenome analysis

The genomic DNA of rhizosphere soil samples was extracted by MagPure Stool DNA KF Kit B (Magen, China), following the manufacturer’s instructions. And the DNA concentration, integrity, and purity were assessed. Subsequently, 1 μg genomic DNA was randomly fragmented by Covaris LE220 (Covaris, Inc., United States). The fragmented DNA was selected by magnetic beads to an average size of 200–400 bp. The selected fragments underwent 3′adenylated, adapters-ligation, PCR amplifying, and the products were purified by the magnetic beads. The double- stranded PCR products were heat denatured and circularized using the splint oligo sequence. The single- strand circle DNA was formatted as the final library. Whole genome sequencing was performed using the MGISEQ- 2000 platform at BGI (Shanghai, China).

Raw data were processed and filtered using Samtools, SOAPnuke, and Bowtie2 software (Langmead and Salzberg, 2012). High-quality clean data were obtained. The samples were *de novo* assembled using the MEGAHIT assembly software. First, MetaGeneMark predicted metagenome genes and eliminated redundancy. Quantification was performed using the Salmon (Buchfink et al., 2014) software, yielding standardized gene abundance values. For non-redundant genes, the BLASTP function of Diamond software annotated functions using databases such as BacMet, CARD, KEGG, eggNOG, COG, Swiss-Prot, and CAZy. Kraken2, with default parameters, performed species annotation in the Nt (202011) database, estimating species-level abundance using a Bayesian algorithm and Kraken classification results with Bracken. Visualization involved analyzing the distribution of genes, species, and functions based on the gene abundance, species abundance, and function abundance tables. Alpha diversity of species, including the Chao1 index, Shannon index, and Simpson index, was calculated using R-packages. LEfSe analysis utilizing the Kruskal-Wallis rank sum test detected differences in species abundance among groups, providing significant characteristics of species-level differences. Pairwise comparisons were performed using Wilcoxon rank sum test. LDA (Linear discriminant analysis) was utilized to estimate LDA scores for the influence of different species on group differences. The Reporter Score method was employed to analyze the enrichment of KEGG pathways with functional differences.

### 2.5 Metabolomics analysis

Ground soil samples (100 mg) were prepared using liquid nitrogen grinding and placed in Eppendorf tubes. Subsequently, 500 μL of 80% methanol aqueous solution was added, followed by vortexing. The sample was allowed to stand on ice for 5 min and then centrifuged at 15,000 *g* at 4°C for 20 min. The obtained supernatant was diluted with LCMS-grade water to achieve a methanol content of 53%. After centrifugation at 4°C for 20 min at 15,000 *g*, the finally obtained supernatant samples were used for LC–MS analysis. Equal volumes of samples were taken from each experimental sample and mixed as QC samples. In the blank samples, 53% methanol aqueous solution was used instead of the experimental samples, and the pretreatment process was the same as that of the experimental samples. Separation was performed on a HypersilGoldcolumn (C18) column at a column temperature of 40°C and a flow rate of 0.2 mL/min. In normal mode, mobile phase A consisted of 0.1% formic acid, whereas mobile phase B consisted of methanol. In the negative mode, mobile phase A was 5 mM ammonium acetate (pH 9.0), and mobile phase B was methanol. The mass spectrometry conditions included a scanning range of m/z 100–1,500. The ESI source parameters were set as follows: spray voltage of 3.5 kV, sheath gas flow rate of 35 psi, auxiliary gas flow rate of 10 L/min, capillary temperature of ion transport tube of 320°C, ion implantation RF level (S-lens RF level) 60, auxiliary gas heater temperature of 350°C, and polarity set to positive/negative. MS/MS secondary scanning was performed by data-dependent scanning.

The identified metabolites were annotated by KEGG database,^1^ HMDB database^2^ and LIPIDMaps database.^3^ The standard of VIP > 1 and *p*-value < 0.05 was used to screen the differential metabolites. The volcano map is drawn with R package ggplot2. Correlation analysis (Pearson correlation coefficient) between different metabolites was carried out by cor in R language, and statistical significance was achieved by cor.mtest in R language, with p-value < 0.05 as statistically significant, and correlation diagram was drawn by corrplot software package in R language. Based on the ChemOnt classification purely based on structure, all known compounds are assigned to more than 4,800 different categories, and then each substance is annotated to four classification levels.

### 2.6 Correlation analysis between microorganisms and differential metabolites

An analysis was conducted to examine the correlation between the bacterial genus-level differences identified through metagenomics and the metabolite differences identified through Metabolomics, using the Pearson correlation coefficient and the R language’s corrplot package to measure the correlation between species diversity and metabolites in environmental samples.

### 2.7 Statistical analysis

The data were recorded and processed using Excel2010 and Statistical Package for the Social Sciences 17.0, with the latter being employed for one-way ANOVA analysis utilizing the least significant difference (LSD) method to determine the significance of differences (*p* < 0.05).

### 2.8 Sequence accession

The datasets presented in this study can be found in online repositories. The names of the repository/repositories and accession number(s) can be found below, Metagenomics: https://www.ncbi.nlm.nih.gov/, PRJNA1051770; Metabolomics: https://www.ebi.ac.uk/metabolights/, MTBLS9186.

## 3 Results and discussion

### 3.1 Effect of γ-PGA application on soil mechanical composition and aggregate ratios

**Figure 1:**
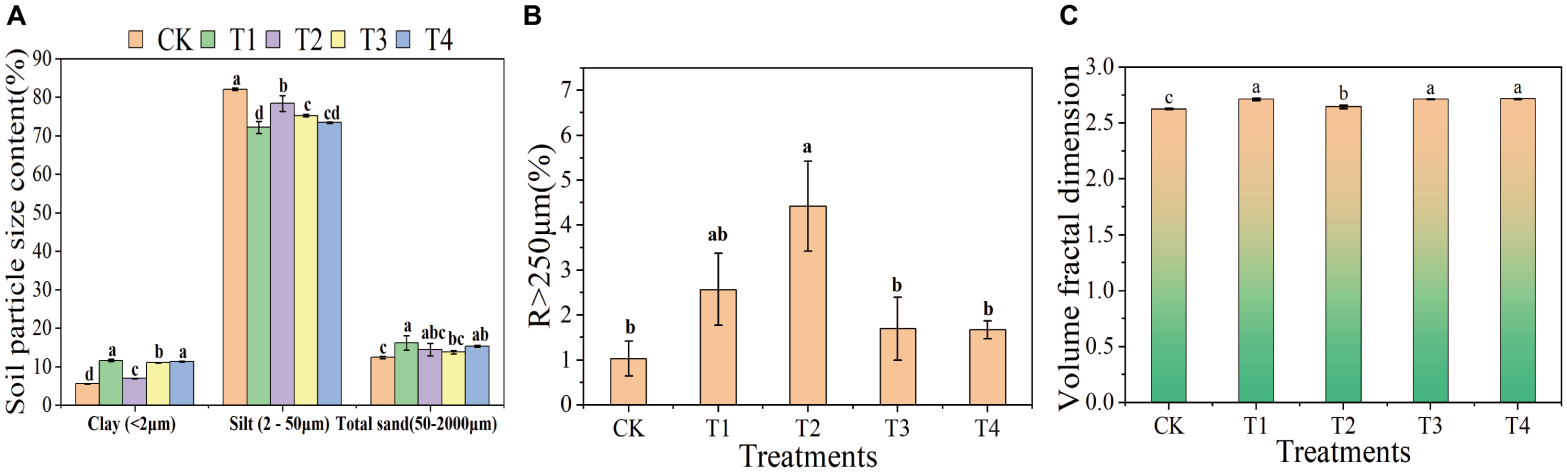
Effects of different γ-PGA treatments on soil texture and structure under drought conditions. **(A)** Soil texture; **(B)** soil aggregates; **(C)** volume classification dimension D of soil particles.

The composition of soil mechanics refers to the various particle components found in soil, including clay, silt, and sand. The ratio and combination of the three main components affect the soil texture, drainage, ventilation, water retention capacity, and other physical properties. This study indicated that compared to CK, the soil with γ-PGA had a decrease in in silt and an increase in clay and sand. Compared to the CK group, the ratio of silt in the T1, T2, T3, T4 group decreased by 12.05, 4.51, 8.33, and 10.55%, respectively. In terms of clay, T1 increased by 1.09 times, T2 increased by 0.25 times, T3 increased by 0.99 times, and T4 increased by 1.04 times compared to the CK group. In terms of sand, T1 increased by 30.94%, T2 increased by 16.24%, T3 increased by 10.99%, and T4 increased by 23.34% compared to the CK group (Figure 1A). Moreover, the proportion of soil micro-aggregates (R < 250 μm) applied with γ-PGA decreased compared to CK, while the proportion of soil macro-aggregates (R > 250 μm) increased. T2 had the greatest increase, accounting for 3.29 times, followed by T1, which increased by 1.5 times, and T3 and T4 increased by 0.65 times and 0.62 times, respectively (Figure 1B).

The study of soil mechanical composition serves as the foundation for investigating various physical and chemical behaviors of soil, which in turn impact soil fertility, water retention, heat, and soil structure (Upadhyay and Raghubanshi, 2020). Previous research by Lagomarsino et al. (2011) and others has demonstrated the impact of adding soil improvers to the soil on its mechanical composition. This study revealed that γ-PGA, as a soil improver, significantly altered the mechanical composition of the soil, resulting in a decrease in the powder content of the soil mechanical composition and an increase in clay and sand content, which was consistent with the findings of Guo et al. (2019).

### 3.2 Effect of γ-PGA application on the fractal dimension of the soil volume

Fractal dimension of the soil volume is an important index to describe the complexity of soil structure. It is calculated according to the soil particle size distribution, which reflects the characteristics of soil particle size distribution and can reveal the structural characteristics and pore distribution of soil. The higher the fractal dimension of the soil volume, the more complex and diverse the pore structure, indicating the presence of more irregularly shaped and sized pores. This type of soil typically exhibited superior water retention and gas-exchange capabilities. Conversely, a lower fractal dimension demonstrated a simpler pore structure, with more uniform and evenly distributed pores. The fractal dimension of soil volume provided insights into the complexity of the soil pore structure. This information was valuable for assessing and managing the soil properties related to water conservation, aeration, and nutrient transport. Compared to CK, the soil volume classification dimension of the γ-PGA treatment group showed significant improvement, particularly in T1, T3, and T4. The improvement in T2 was the least, whereas there were differences. The volume classification dimension increased by 3.30, 0.74, 3.31, and 3.41% in T1, T2, T3, and T4, respectively, compared to CK (Figure 1C).

The larger dimension of soil volume classification was associated with more uniform soil texture (Wang et al., 2022). This relationship was attributed to the incorporation of γ-PGA, which enhanced the proportion of soil mechanical composition and consequently increased the dimension of soil volume classification compared to CK.

### 3.3 Effect of γ-PGA on soil infiltration characteristics

**Figure 2:**
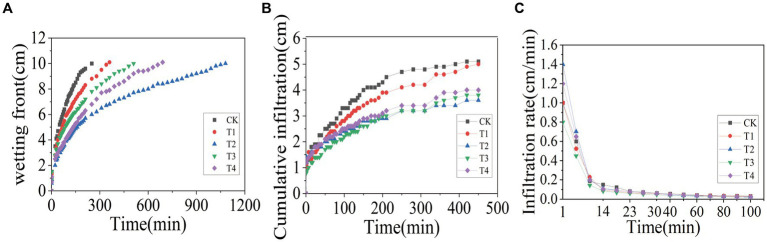
Effects of various γ-PGA treatments on soil infiltration characteristics under drought conditions. **(A)** Wetting front; **(B)** cumulative infiltration; **(C)** infiltration rate.

In order to investigate the effects of γ-PGA application on the soil infiltration characteristics, one-dimensional soil column was used for vertical infiltration tests. The results, presented in Figure 2A, showed that under the condition of controlling the wetting peak to be 10 cm, the CK group arrived first, lasting for 250 min, followed by the T1 group, lasting for 350 min; the T3 group, lasting for 510 min; the T4 group, lasting for 690 min; and the T2 group, lasting for 1,080 min. According to Figure 2B, when the CK group reached the end of the wetting peak of 10 cm, the cumulative infiltration of the CK group was 4.7 cm, while under the same duration, the cumulative infiltration of the γ-PGA-added groups was 4.1 cm (T1), 3.2 cm (T2), 3.2 cm (T3), and 3.4 cm (T4), respectively. Compared to the CK treatment, it decreased by 12.77% (T1), 31.91% (T2), 31.91% (T3), and 27.66% (T4). Figure 2C illustrates that with the passage of the soil column infiltration time, the soil infiltration rate tended to be stable after 60 min. When the infiltration time was 60 min, soil water infiltration rates were 0.043 cm/min (CK), 0.038 cm/min (T1), 0.035 cm/min (T2), and 0.03 cm/min, respectively. Compared with the CK treatment, it decreased by 11.54% (T1), 19.23% (T2), 30.77% (T3), and 15.38% (T4), respectively.

The soil infiltration rate, as a crucial parameter of soil moisture characteristics, reflects soil permeability and water retention capacity. As reported by Guo et al. (2021), the application of a specific proportion of γ-PGA to soil can enhance the infiltration characteristics of soil, which was consistent with the results of this study. In this study, the initial infiltration rate of T2 was the highest, which may be attributed to the increased proportion of large aggregates in the T2 group, resulting in larger pores and a more rapid initial infiltration rate. Once the pores were filled with water and reached a stable level, the infiltration rate returned to the normal level.

### 3.4 Effects of γ-PGA on basic physical and chemical properties of soil

Three biological replicates of each treatment group were selected to assess the fundamental physical and chemical properties of the soil. The results presented in Table 1 demonstrated that the bulk density of the γ-PGA-treated group, specifically the T4 group, was significantly lower than that of the CK group, with the largest decrease measured at 4.03%. Compared with CK, there was no significant difference in pH value in the γ-PGA treated group. Compared with CK, the electrical conductivity of T3 group was significantly improved by 1.36 times, and that of T1, T2, and T4 were slightly improved (7.04, 5.68, and 18.22% respectively). Compared with CK, the organic matter in T4 group increased by 56.32%, while that of T1, T2, and T3 did not change significantly, with the increasing ranges of 6.70, 5.51, and 9.07%, respectively. Furthermore, the activities of three enzymes representative of soil fertility were significantly improved. The urease activity in the T3 and T4 groups significantly increased by 18.91 and 15.63%, respectively. The T1 and T2 groups also experienced increases, while the difference was not statistically significant. Similarly, sucrase activity significantly improved only in the T4 group, which was 28.37% higher than that in the CK group. The alkaline phosphatase activity in the T3 group was the highest, reaching 14766.15 U/g, followed by the T2 group (9837.00 U/g), the T1 group (7739.72 U/g), and the T4 group (4950.44 U/g). Overall, this study indicated that the application of γ-PGA had a positive impact on the basic fundamental physical and chemical properties of soil under drought conditions.

**Table 1:** Effects of γ-PGA treatment on soil properties under drought conditions.

	CK	T1	T2	T3	T4
Volumetric weight (g/cm^3^)	1.55 ± 0.02^a^	1.54 ± 0.01^a^	1.52 ± 0.02^ab^	1.54 ± 0.03^b^	1.49 ± 0.02^b^
pH	6.98 ± 0.06^a^	7.09 ± 0.02^a^	7.07 ± 0.09^a^	7.19 ± 0.32^a^	7.03 ± 0.12^a^
Conductivity (us/cm)	152.03 ± 8.64^b^	162.73 ± 44.89^b^	160.67 ± 6.56^b^	358.67 ± 14.01^a^	179.73 ± 18.06^b^
Organic matter (g/kg)	1.43 ± 0.13^b^	1.53 ± 0.09^b^	1.51 ± 0.08^b^	1.56 ± 0.16^b^	2.24 ± 0.16^a^
Ureasemg/(g*d)	6.26 ± 0.17^b^	6.65 ± 0.78^ab^	6.81 ± 0.66^ab^	7.44 ± 0.56^a^	7.24 ± 0.20^a^
S-SCmg/(g*d)	13.37 ± 0.60^b^	14.10 ± 1.50^b^	14.73 ± 0.25^ab^	15.19 ± 2.84^ab^	17.16 ± 1.22^a^
S-AKP (U/g)	3821.95 ± 871.94^c^	7739.72 ± 462.10^bc^	9837.00 ± 4298.29^b^	14766.15 ± 2870.96^a^	4950.44 ± 1678.81^c^

Data represent mean ± standard deviation (SD). LSD test was used to assess the differences. Different lowercase letters denote significant differences between the treatments (*p* < 0.05).

Numerous studies have demonstrated that soil properties, including pH, bulk density, organic matter, and enzyme activity, serve as comprehensive indicators of the effects of soil amendments (Fan et al., 2022). Notably, the effect of γ-PGA on soil pH was negligible. Augmenting soil organic matter content has been consistently linked to enhanced enzyme activities, specifically urease, sucrase, and alkaline phosphatase, which are indicative of improved soil fertility (Zhang et al., 2017; Yin et al., 2018), consistent with the findings of this study. Furthermore, γ-PGA has proven effective in reducing soil bulk density, establishing its efficacy as a soil conditioner (Garbowski et al., 2023). Changing the mechanical composition of the soil within an optimal range enhanced soil texture. This led to the increased formation of large aggregates, improving soil aeration and water retention. The observed outcomes aligned with the properties of clay particles, known for their large surface area that enhanced water retention, and sand particles, which contributed to the formation of larger pores that facilitated air circulation and rapid water drainage. This enhanced the fertility of the soil, which is concomitant with the augmentation of organic matter and the activities of three enzymes indicative of soil fertility. Hence, the incorporation of γ-PGA into the soil can yield numerous advantages, improve the texture and properties of the soil under arid conditions, facilitate the enhancement of aeration, water retention, and fertility, and subsequently engender a healthier and more productive soil. This is of paramount importance for agricultural production, soil conservation, and sustainable development.

### 3.5 Analysis of soil microbial structure composition

**Figure 3:**
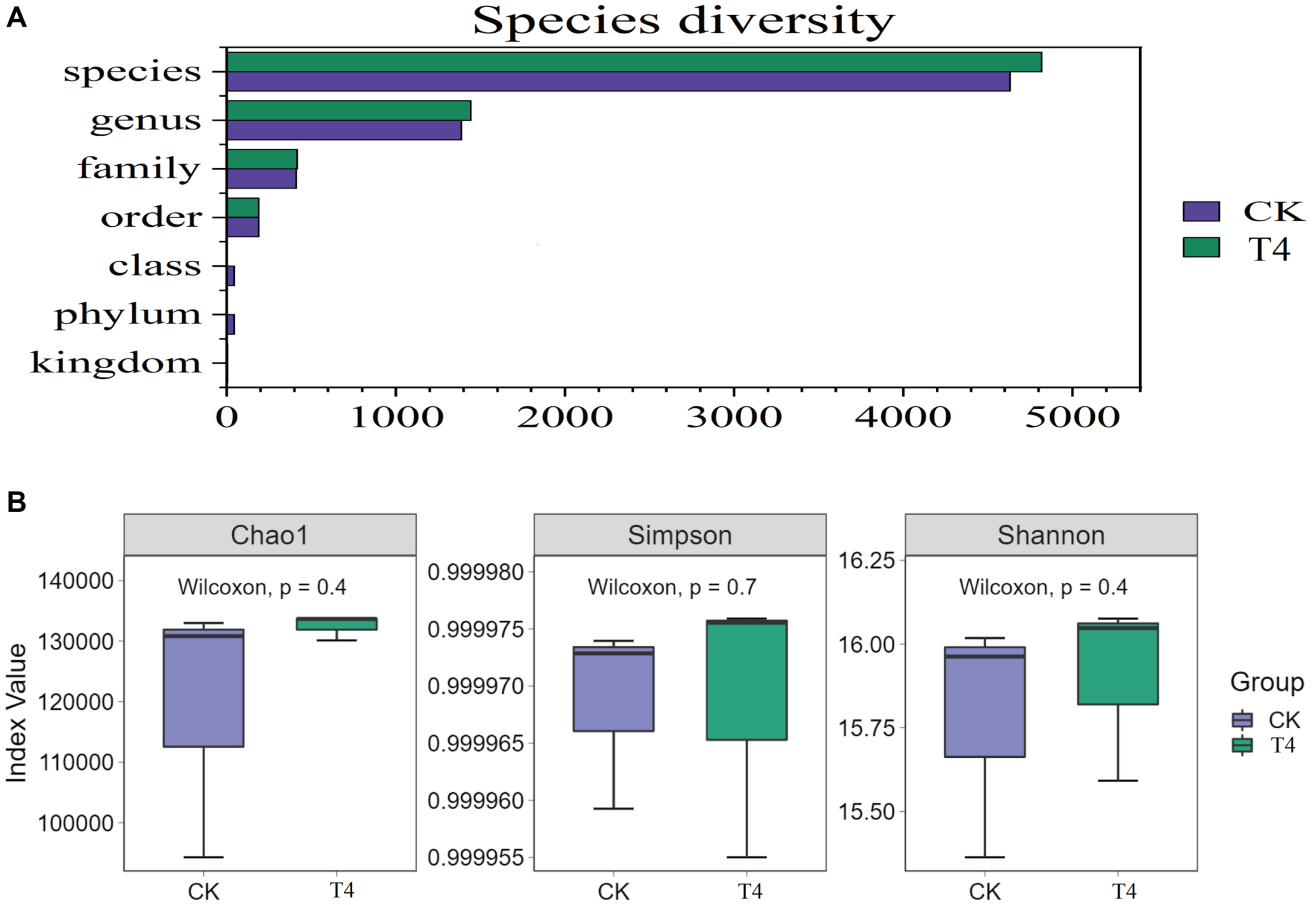
Genetic diversity analysis of metagenomes treated with γ-PGA under drought conditions. **(A)** Composition of the soil microbial community; **(B)** box diagram of alpha diversity.

**Figure 4:**
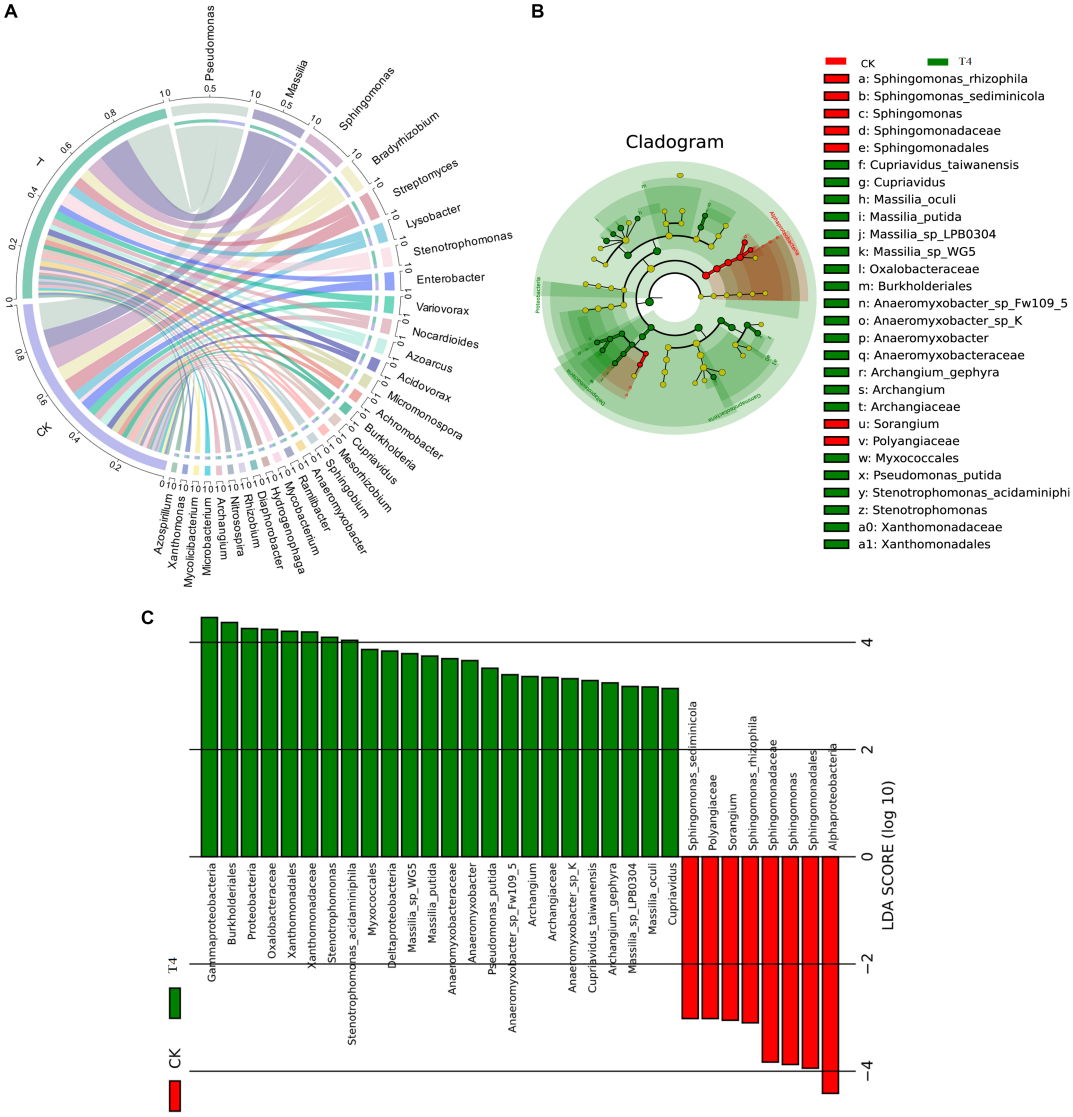
Species analysis of metagenomes treated with γ-PGA under drought conditions. **(A)** Circos diagram of the top 30 dominant genera aggregated at the genus level. The circle is divided into left and right parts, indicating the grouping information on the left and species classification information on the right. Moving from the outside circle to the inside, the scale of the outermost circle represents the proportion of species in the grouping, or the proportion of different species in the grouping. The color of the arc segment of the inner circle indicates the grouping/sample or species, whereas the connecting lines between the arc segments indicates the presence of species in the sample. The width of the connecting line on the arc segment indicates its proportion. **(B)** Top 30 LEfSe circular evolutionary branching graphs at the species level. The evolutionary branching graph is circular, comprising multiple rings, with the inner ring at a high taxonomic level and the outer ring at a low taxonomic level. Each point represents a specific species classification, with size representing relative abundance. Species with no significant differences are marked in yellow, whereas taxa with significant differences are colored according to the groups. **(C)** Histogram of LDA value distribution of the first 30 LEfSe LDA values at different levels. The LDA value exceeds the set threshold (usually 2). The ordinate represents the classification unit with differences, and the abscissa represents the LDA value. The greater the LDA value, the more significant is its contribution to the differences between the groups. The color of the columns indicates grouping, with positive and negative values in the abscissa indicating the direction.

Because T4 group is in a stable state of promoting improvement in all basic physical and chemical properties of soil. Based on this result, CK group and T4 group, the most representative treatment group, were selected for soil metagenome and metabolomics analysis. After high-throughput sequencing of soil samples from the different treatments, the composition and diversity of the soil microbial community were determined. A total of 7,423 OTU was obtained through sequencing, representing 5,133 species across 1,519 genera, 197 orders, 431 families, and 45 phyla (Figure 3A). The results indicated that the Chao1, Shannon, and Simpson indices of the γ-PGA-treated group were higher than those of the CK group, although the difference was not statistically significant (Figure 3B). At the genus level, the top 20 dominant genera of maize rhizosphere aggregation in the γ-PGA treatment group and non-γ-PGA treatment group, including *Pseudomonas*, *Massilia, Sphingomonas, Bradyrhizobium, Streptomyces, Lysobacter, Stenotrophomonas, Enterobacter,* Var*iovorax, Nocardioides, Azoarcus, Acidovorax, Micromonospora, Achromobacter, Burkholderia, Cupriavidus, Mesorhizobium, Sphingobium, Anaeromyxobacter,* and *Ramlibacter*. Compared with the CK group, the relative abundances of *Pseudomonas, Massilia, Lysobacter, Stenotrophomonas, Acidovorax, Achromobacter, Cupriavidus, Anaeromyxobacter*, and *Ramlibacter* increased significantly. In particular, *Stenotrophomonas, Anaeromyxobacter, Massilia,* and *Pseudomonas* increased by 2.67 times, 2.02 times, 0.71 times, and 0.64 times, respectively (Figure 4A). These findings suggested that γ-PGA application affected the structural composition and proportion of soil microorganisms.

Considering the absence of inter-group discrimination analysis, the LEfSe method was employed to examine differences in abundance between species across the two groups. A total of 28 biomarker species were identified, comprising 7 species in the CK group and 21 species in the T group (Figure 4B). *Alphaproteobacteria* exhibited the highest abundance in the CK group, based on the LDA score (log10) > 4. In the T group, eight species with significant differences played a pivotal role, namely *Garhmaproteobacteria*, *Burkholderiales*, *Proteobacteria*, *Oxalobacteraceae*, *Xanthomonadales*, *Xanthomonadaceae*, *Stenotrophomonas*, and *Stenotrophomonas acidaminiphila* (Figure 4C).

The soil metagenome can be utilized to not only evaluate the structure and diversity of the soil microbial community but also to identify meaningful microbial genera and predict their metabolic functions (Li et al., 2020). This approach can provide comprehensive insights into the response to soil conditioners and reflect changes in the physical and chemical properties of the soil. Yu et al. (2022) found that γ-PGA had a modest impact on the structure and diversity of the soil microbial community, which mainly affected the alteration of the soil bacterial community and changed the relative abundance at different classification levels. *Proteobacteria*, *Actinobacteria*, and *Acidobacteria* predominated the soil bacterial community and played a favorable role in soil improvement. This is consistent with the results of this study, which revealed that Proteobacteria, Actinobacteria, Bacteroidetes, and *Acidobacteria* were the dominant enriched phyla. Additionally, *Pseudomonas, Massilia, Lysobacter, Azoarcus, Cupriavidus*, and *Anaeromyxobacter*, which exhibited significantly increased relative abundances, played a crucial role in soil nitrogen fixation (Yu et al., 2022). Concurrently, numerous soil microbial communities can dissolve insoluble phosphorus complexes into soluble forms that can be easily absorbed by plants. *Bacillus* and *Pseudomonas* are the most common phosphate-solubilizing bacteria (PSB) (Goyal et al., 2021). It was reported Pseudomonas could help the plant to produce osmoreceptors (proline, choline, and glycine betaine), and photosynthetic pigments and reduced the production of MDA contents to mitigate the adverse effects of the moisture stress (Yasmin et al., 2022). *Azoarcus* has the ability to dissolve inorganic phosphate (Fernández-Llamosas et al., 2021), whereas *Massilia* is known to be rich in the plant rhizosphere and is involved in the soil carbon cycle, particularly in the colonization of roots (Raths et al., 2019). Krishnamoorthy et al. found that the combined application of *Massilia* and arbuscular mycorrhizal fungi alleviated the effects of salt stress on plant growth, root colonization, nutrient accumulation and growth (Krishnamoorthy et al., 2016). Turnbull et al. reported that isolated a strain of *Massilia* could produce heteroauxin and cellulose-degrading enzymes to promote plant growth (Turnbull et al., 2012). Furthermore, *Azoarcus*, *Massilia*, and *Anaeromyxobacter* have been shown to participate in the formation of soil aggregates through the secretion of biological cells and production of adhesive molecules. *Anaeromyxobacter* is globally distributed in soil environments, its importance as a diazotroph in nature has been confirmed (Masuda et al., 2020). These aggregates are important for the stable conservation of the soil structure and the formation and maintenance of nutrients (Vinatzer et al., 2012; Fernández-Llamosas et al., 2021; Song et al., 2022). This is consistent with the finding that the addition of γ-PGA to soil can improve the proportion and fertility of soil macro-aggregates.

**Figure 5:**
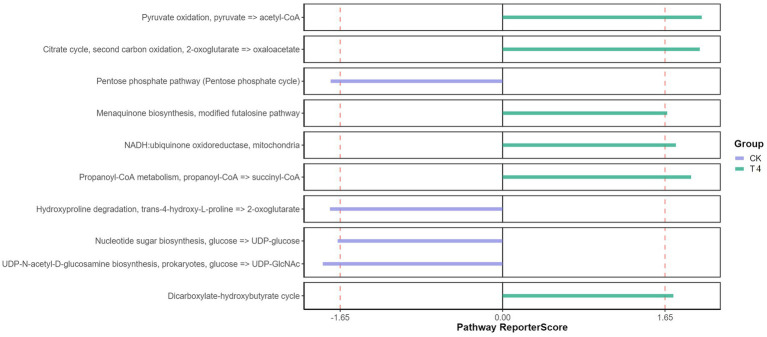
Functional KEGG pathway enrichment pathway diagram. The Reporter score threshold of significant level was 1.65, and the positive and negative values indicate the enrichment direction. The plus sign indicates the enrichment in the right group.

Furthermore, the KEGG pathway database was utilized to predict function, compared with the CK group, the T experimental group demonstrated significantly enriched pathways, including Pyruvate oxidation, Citrate cycle, Menaquinone biosynthesis, NADH, propanoyl-CoA metabolism, and dicarboxylate-hydroxybutyrate cycle (Figure 5). Pyruvate oxidation, Citrate cycle, and Menaquinone biosynthesis are significant in releasing energy for microbial life activities (Su et al., 2018). The TCA cycle is also the final metabolic pathway and hub for the three main nutrients (sugar, lipid, and amino acids). This enrichment may be attributed to the decrease in soil moisture and organic matter secreted by plant roots under drought treatment, which forces microorganisms to obtain energy more efficiently to adapt to drought environments. These enriched pathways provide additional metabolic pathways and generate extra energy, allowing microorganisms to maintain their activities (Li et al., 2022). The menaquinone biosynthesis and NADH pathways also involve electron transfer processes, which play a crucial role in the redox reactions of microorganisms (Dhiman et al., 2009). This enrichment may be due to the lack of oxygen supply in the soil under drought conditions, and microorganisms must initiate and participate in redox reactions to adjust the redox balance in cells (de Vries et al., 2020). Enrichment of certain metabolic pathways may aid microorganisms in coping with oxygen limitation and oxidative stress in arid environments. The dicarboxylate-hydroxybutyrate and Propanoyl-CoA metabolic pathways are particularly important for substrate utilization and metabolism. In arid environments, drought can lead to a decrease in soil moisture and limit organic matter availability. These enriched pathways provide a means for microorganisms to decompose and utilize substrates that can promote survival and activity under such conditions (Li et al., 2022). Overall, the enrichment of these pathways in microorganisms under drought conditions may help them adapt to and respond to the challenges of the drought environment. By enhancing energy production, regulating redox balance, and providing substrate utilization, microorganisms can better survive and maintain their life activities, thereby playing an important role in the function and stability of the soil ecosystem.

Therefore, the utilization of γ-PGA in soil can be beneficial in terms of the predominance of carbon, nitrogen, and phosphorus cycling, which positively affected the biochemical cycling of soil. This improvement in the physical and chemical properties of the soil contributed to a better rhizosphere environment, ultimately playing a crucial role in enhancing the adaptability and productivity of plants in adverse environments.

### 3.6 Metabolic differences of soil treated with γ-PGA

**Figure 6:**
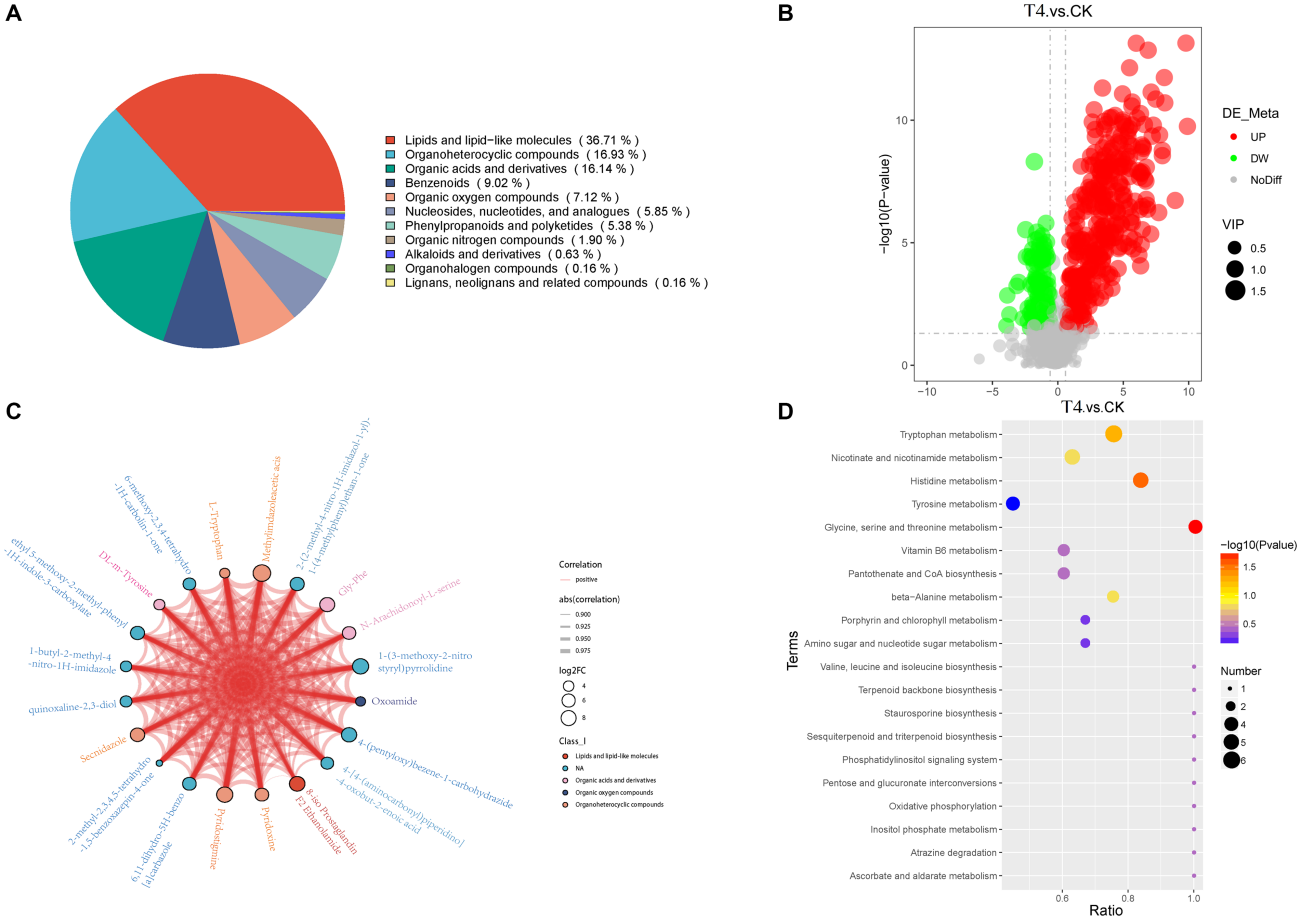
Metabonomic analysis of soil treated with γ-PGA under drought conditions. **(A)** Pie chart of metabolite Class I classification. **(B)** Volcano map of differential metabolites. The abscissa represents the log2(Fold Change) of metabolites in different groups, whereas the ordinate represents the significance level [−log10(*p*-value)]. Each point on the volcanic map represents a metabolite, with significantly up-regulated metabolites depicted by red dots and significantly down-regulated metabolites represented by green dots. The size of each dot represents a VIP value. **(C)** Differential metabolites and chordogram. The top 20 differential metabolites were selected for display in the chord diagram. The color of the dots represents different kinds of metabolites, with the size representing log2(Fold Change). The thickness of the connecting lines between the metabolites represents the correlation, where blue lines represent negative correlations, and red lines represent positive correlations. **(D)** KEGG enriched bubbles legend. The abscissa in the figure is x/y (the number of differential metabolites in the corresponding metabolic pathway/ total number of metabolites identified in this pathway). The color of the point represents the *p*-value of the hypergeometric test, where smaller values indicate greater reliability and statistical significance. The size of the dots represents the number of differential metabolites in the corresponding pathway, with larger dots indicating more differential metabolites in the pathway.

Using non-targeted metabolomics technology, alterations in metabolite levels of soil samples form CK, T4 group were investigated, resulting in the identification of 632 metabolites across 12 samples, which were categorized into 11 groups (Figure 6A). These groups comprised lipids and lipid-like molecules (232, 36.71%), organoheterocyclic compounds (107, 16.93%), organic acids and derivatives (102, 16.14%), benzenoids (57, 9.02%), organic oxygen compounds (45, 7.12%), nucleosides, nucleotides, and analogs (37, 5.85%), phenylpropanoids and polyketides (4, 5.38%), organic nitrogen compounds (12, 1.90%), alkaloids and derivatives (4, 0.63%), organohalogen compounds (1, 0.16%), and lignans, neolignans, and related compounds (1, 0.16%). Based on the VIP > 1.0, and *p*-value < 0.05, 456 metabolites were detected in the γ-PGA experimental group, among which 340 were up-regulated and 116 were down-regulated, indicating an overall up-regulated trend (Figure 6B). Furthermore, visualization of the correlation and chord diagram of NA (six species) revealed that although the difference between multiple compounds was substantial, the trend of metabolite changes was similar and displayed a high positive correlation (Figure 6C).

**Figure 7:**
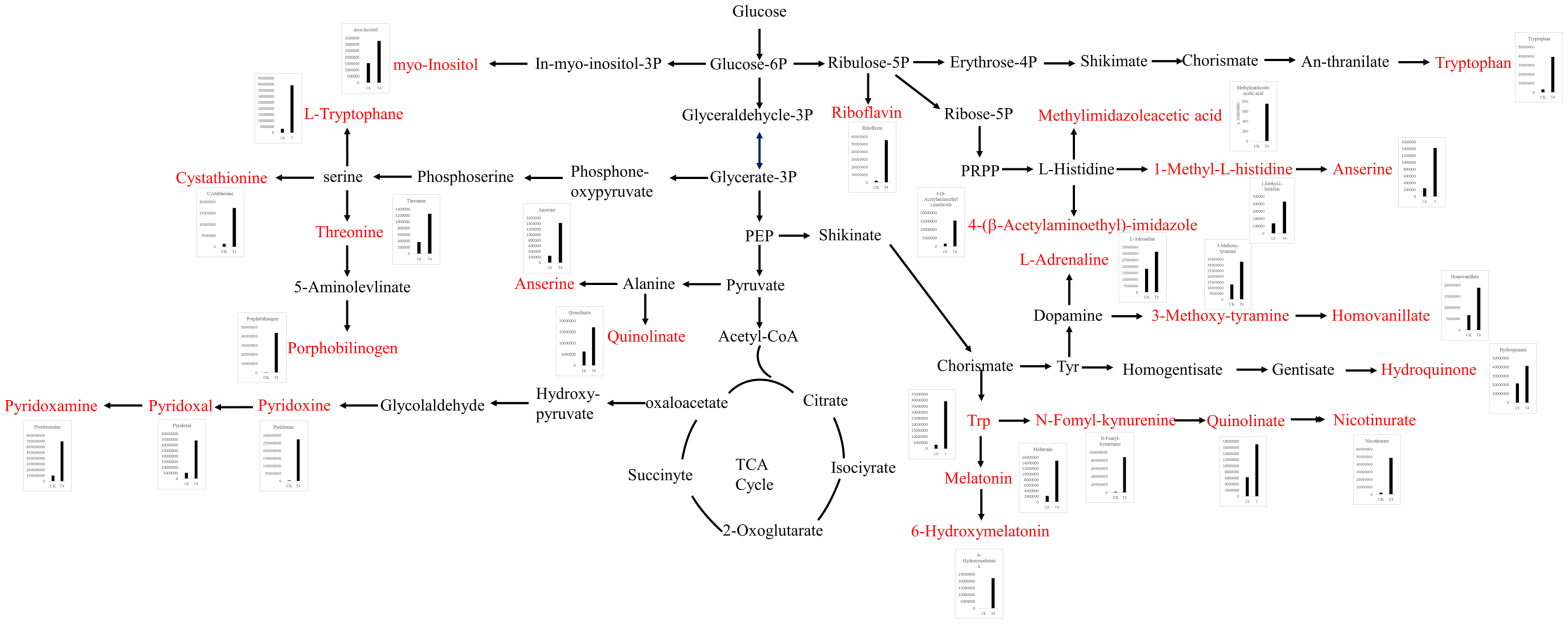
Metabolic pathway map of differentially up-regulated marker metabolites in soil treated with γ-PGA under drought conditions.

The differential metabolites were annotated in the KEGG database, and the top 10 enriched metabolic pathways identified included glycine, serine, and threonine metabolism, histidine metabolism, tryptophan metabolism, beta-alanine metabolism, nicotinate and nicotinamide metabolism, Vitamin B6 metabolism, pantothenate and CoA biosynthesis, pentose and glucuronate interconversions, ascorbate and aldarate metabolism, and oxidative phosphorylation (Figure 6D). A total of 22 up-regulated differential metabolic compounds were involved in the enriched metabolic pathways, including methylimidazole-acetic acid, pyridoxamine, pyridoxine, N-formyl-kynurenine, riboflavin, porphobilinogen, L-adrenaline, hydroquinone, tryptophan, homovanillate, nicotinurate, 3-methoxy-tyramine, pyridoxal, 6-hydroxymelatonin, quinolinate, cystathionine, 4-(b-acetylaminoethyl)-imidazole, melatonin, myo-inositol, anserine, threonine, and 1-methyl-L-histidine. The levels of these metabolites were significantly different between the groups, and were more abundant in PGA treated group. The differentially up-regulated metabolites and their abundance were sorted and classified into metabolic maps (Figure 7).

Soil non-targeted metabolomics culminates in alterations in soil fertility and microbial diversity (Swenson et al., 2015). Metabolites may significantly impact the microbial composition of rhizosphere soil, as they can determine microbial food webs, regulate soil chemistry, modify microbial gene expression, and even serve as symbolic chemicals to mediate microbial-microbial interactions (Hu et al., 2018). Currently, there have been few reported studies on soil metabolomics utilizing γ-PGA. In this study, the soil metabolites of maize plants were analyzed, revealing that the dominant metabolites were lipids, lipid-like molecules, organic compounds, and organic acids and derivatives, comprising approximately 70% of the total metabolites. These metabolites primarily comprised nutrients necessary for the growth of maize and soil microorganisms, with a significant enrichment in the amino acid and vitamin metabolism pathways. Amino acids are typically the second most abundant components of root exudates, and can provide carbon skeletons and amino nitrogen for microorganisms (Jaeger et al., 1999). Tahoun et al. reported that humic acid, a soil improver, and zinc oxide nanoparticles and tryptophan, two plant growth stimulators, were applied to nutrient-poor sandy soil through foliar and soil application of wheat, respectively. This treatment resulted in rapid formation of large aggregates, reduced bulk density and pH value, increased porosity and conductivity, and improved soil structure. These improvements in the soil hydraulic characteristics have led to positive results for both soil and wheat crops (Tahoun et al., 2022). Pyridoxine and riboflavin, two essential vitamins, serve as nutrients for *Cellulomonas, Rhodococcus, Pseudomonas, Bacillus,* and *Arthrobacter*, enhancing their growth and leading to the increased consumption of hydrocarbons, biotin, and other vitamins (Al-Mailem et al., 2013). Consequently, the application of γ-PGA to soil, particularly under drought conditions, altered the metabolic landscape of the soil and affected the types and concentrations of various chemical metabolites. This not only positively affected the physical and chemical properties of the soil, creating a conducive environment, but also provided essential nutrients for microbial activities. This, in turn, promoted the growth and reproduction of microorganisms, contributing to the diversity and functionality of the soil microbial communities.

### 3.7 Correlation analysis between microbial genera and soil metabolites

**Figure 8:**
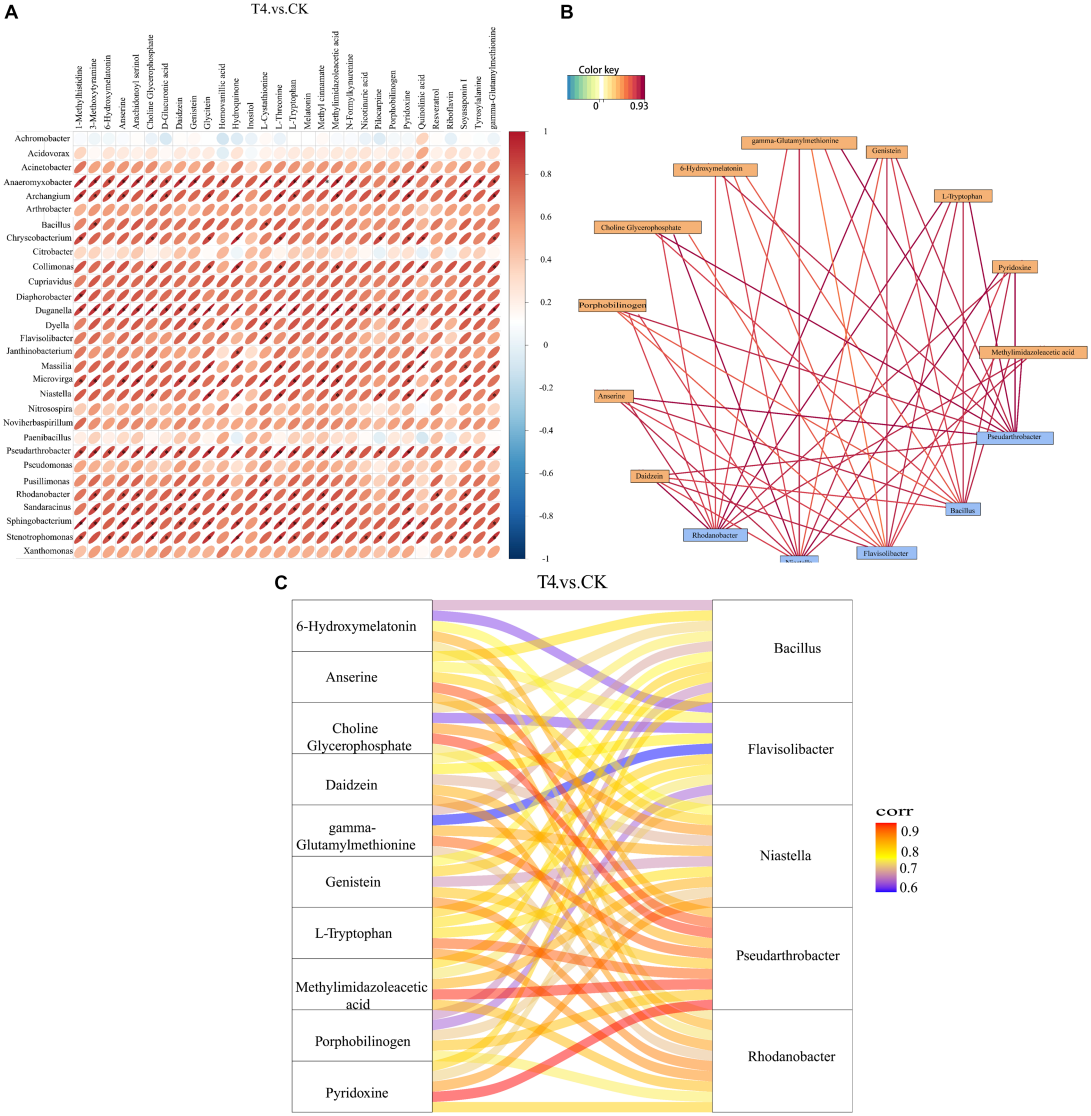
Correlation analysis between different genera and different metabolites at top 30 level. **(A)** Correlation analysis heat map. The horizontal direction represents different bacteria, and the vertical direction represents different metabolites. The legend on the right side indicates the correlation coefficient. The color spectrum ranges from red (strong positive correlation) to blue (strong negative correlation), with the intensity of the color representing the strength of the correlation. The flatter the ellipse, the higher the absolute value of the correlation. The parts marked with an asterisk (*) in the result map indicate statistical significance at *p* ≤ 0.05. **(B)** Correlation analysis network diagram. Yellow boxes represent metabolites, and blue boxes represent bacteria. Red lines indicate positive correlations, while blue lines indicate negative correlations. **(C)** Correlation analysis of Sangji diagram. The left side shows differential metabolites, and the right side displays differential bacteria. The connecting lines represent correlations, where red and blue lines indicate positive and negative correlations, respectively.

The correlation analysis between the bacteria with significant difference in genus level obtained by metagenomics analysis and the metabolites with significant difference obtained by metabolomics analysis was based on Pearson correlation coefficient, and a thermal map was obtained (Figure 8A). The results revealed that the total number of 30 genera demonstrated a strong correlation with most of the differential metabolites, indicating that these microorganisms might participate in the formation of the majority of the metabolites in the soil. Subsequently, the potential correlation between key bacteria and metabolites was investigated, and there were 10 metabolites at the key nodes. Daidzein, anserine, porphobilinogen, choline-glycerophosphate, 6-hydroxymelatonin, gamma-glutamylmethionine, genistein, L-tryptophan, pyridoxine, and methylimidazoleacetic acid showed strong correlations with five genera at key nodes, including *Rhodanobacter*, *Niastella*, *Flavisolibacter*, *Bacillus*, and *Pseudo*-*Arthrobacter* (Figure 8B). The Sangji diagram revealed that up to 6 key metabolites, such as anserine, choline glycerophosphate, gamma-glutamylmethionine, L-tryptophan, methylimidazole acid, and pyridoxine, were strongly correlated with the key genus *Pseudarthrobacter* (Figure 8C).

The correlation analysis between the soil metagenome and metabolomics is crucial for understanding the significance of research on the diversity of microorganisms to macro-metabolites. The bacterial community is the primary driver of soil metabolic changes (Ren et al., 2022), and bioactive metabolites in turn affect soil microbial diversity (Bi et al., 2022). Therefore, this study analyzed the correlation between different metabolites and different bacterial genera (the top 30) between γ-PGA and CK groups. Generally, there was a positive correlation between differential metabolites and different genera. Metabolites at key nodes, such as pyridoxine, daidzein, and choline-glycerophosphate, were strongly correlated with *Rhodanobacter*, *Niastella*, *Flavisolibacter*, *Bacillus*, and *Pseudarthrobacter*. Certain published studies have suggested that *Pseudomonas, Bacillus*, and *Arthrobacter* grow better and consume more hydrocarbons, biotin, and other vitamins when pyridoxine is provided (Al-Mailem et al., 2013). Daidzein is an isoflavone secreted from the soybean root system that has excellent fluidity in soil, participates in the adsorption of soil organic matter in gray lowlands, and also shapes the rhizosphere bacterial community to some extent, assembling beneficial microorganisms to reduce pathogen damage (Okutani et al., 2020). Diverse soil bacteria utilize choline as the only carbon and nitrogen source, and this catabolic pathway may be particularly important for bacteria related to eukaryotes (Nock et al., 2016). Therefore, the results of this study demonstrated that these beneficial bacteria existing in the soil with γ-PGA as core endophytes interact with key metabolites and play an essential ecological role.

## 4 Conclusion

Through extensive examination of metagenome and metabolomics, this study revealed the regulatory impact of γ-PGA on soil characteristics under drought conditions. The γ-PGA had a significant impact on the community structure and metabolite composition of soil microorganisms, which in turn leads to improvements in the soil texture and nutrient content. Furthermore, enhanced soil quality provides a favorable environment for soil microbial communities, enabling them to better adapt to their surroundings through the production of metabolites. These insights offer new perspectives and methods for optimizing soil quality and enhancing the ability of crops to thrive in arid environments. Future research into the molecular mechanisms underlying the regulation of soil characteristics by γ-PGA will deepen our understanding of the function and stability of soil ecosystems, providing a scientific basis and technical support for agricultural production in arid regions.

## Data availability statement

The datasets presented in this study can be found in online repositories. The names of the repository/repositories and accession number(s) can be found at: https://www.ncbi.nlm.nih.gov/, PRJNA1051770; https://www.ebi.ac.uk/metabolights/, MTBLS9186.

## Author contributions

LH: Formal analysis, Investigation, Methodology, Writing – original draft. LW: Funding acquisition, Supervision, Writing – review & editing. GF: Supervision, Writing – review & editing. LJ: Data curation, Software, Writing – review & editing. TS: Formal analysis, Investigation, Writing – review & editing. YH: Data curation, Formal analysis, Writing – review & editing. RY: Project administration, Writing – review & editing. GX: Data curation, Formal analysis, Writing – review & editing. YC: Data curation, Formal analysis, Writing – review & editing.

## Footnotes

^1^ https://www.genome.jp/kegg/pathway.html

^2^ https://hmdb.ca/metabolites

^3^ http://www.lipidmaps.org/

## References

[ref1] Abram F. (2015). Systems-based approaches to unravel multi-species microbial community functioning. Comput. Struct. Biotechnol. J. 13, 24–32. doi: 10.1016/j.csbj.2014.11.009 25750697 PMC4348430

[ref2] Al-Mailem D. M. Eliyas M. Radwan S. (2013). Enhanced bioremediation of oil-polluted, hypersaline, coastal areas in Kuwait via vitamin-fertilization. Environ. Sci. Pollut. Res. 21, 3386–3394. doi: 10.1007/s11356-013-2293-6 24243095

[ref3] Ault T. R. (2020). On the essentials of drought in a changing climate. Science 368, 256–260. doi: 10.1126/science.aaz5492 32299944

[ref4] Bi B. Yuan Y. Zhang H. Wu Z. Wang Y. Han F. J. A. S. E. (2022). Rhizosphere soil metabolites mediated microbial community changes of *Pinus sylvestris* var. mongolica across stand ages in the mu Us Desert. Appl. Soil Ecol. 169:104222. doi: 10.1016/j.apsoil.2021.104222

[ref5] Buchfink B. Xie C. Huson D. H. (2014). Fast and sensitive protein alignment using DIAMOND. Nat. Methods 12, 59–60. doi: 10.1038/nmeth.3176 25402007

[ref6] Chang F. Li W. Hu H. Ge F. Chen G. Ren Y. (2022). Chemical pretreatment and saccharification of corncob for poly-γ-glutamic acid production by *Bacillus subtilis* SCP010-1. Process. Saf. Environ. Prot. 159, 1184–1193. doi: 10.1016/j.psep.2022.01.071

[ref7] Chen L. Su W. Xiao J. Zhang C. Zheng J. Zhang F. (2021). Poly-gamma-glutamic acid bioproduct improves the coastal saline soil mainly by assisting nitrogen conservation during salt-leaching process. Environ. Sci. Pollut. Res. Int. 28, 8606–8614. doi: 10.1007/s11356-020-11244-7 33063212

[ref8] de Vries F. T. Griffiths R. I. Knight C. G. Nicolitch O. Williams A. (2020). Harnessing rhizosphere microbiomes for drought-resilient crop production. Science 368, 270–274. doi: 10.1126/science.aaz5192 32299947

[ref9] Dhiman R. K. Mahapatra S. Slayden R. A. Boyne M. E. Lenaerts A. Hinshaw J. C.. (2009). Menaquinone synthesis is critical for maintaining mycobacterial viability during exponential growth and recovery from non-replicating persistence. Mol. Microbiol. 72, 85–97. doi: 10.1111/j.1365-2958.2009.06625.x 19220750 PMC4747042

[ref10] Duan M. Li Y. Zhu G. Wu X. Huang H. Qin J.. (2023). Soil chemistry, metabarcoding, and metabolome analyses reveal that a sugarcane—Dictyophora indusiata intercropping system can enhance soil health by reducing soil nitrogen loss. Front. Microbiol. 14:1193990. doi: 10.3389/fmicb.2023.1193990 37303785 PMC10249477

[ref11] Fan Y. Liu J. Liu Z. Hu X. Yu Z. Li Y.. (2022). Chitin amendments eliminate the negative impacts of continuous cropping obstacles on soil properties and microbial assemblage. Front. Plant Sci. 13:1067618. doi: 10.3389/fpls.2022.1067618 36507440 PMC9730418

[ref12] Fernández-Llamosas H. Díaz E. Carmona M. (2021). Motility, adhesion and c-di-GMP influence the endophytic colonization of rice by *Azoarcus* sp. CIB. Microorganisms 9:554. doi: 10.3390/microorganisms9030554 33800326 PMC7998248

[ref13] Garbowski T. Bar-Michalczyk D. Charazińska S. Grabowska-Polanowska B. Kowalczyk A. Lochyński P. J. S.. (2023). An overview of natural soil amendments in agriculture. Soil Tillage Res. 225:105462. doi: 10.1016/j.still.2022.105462

[ref14] Goyal R. K. Schmidt M. A. Hynes M. F. (2021). Molecular biology in the improvement of biological nitrogen fixation by rhizobia and extending the scope to cereals. Microorganisms 9:125. doi: 10.3390/microorganisms9010125 33430332 PMC7825764

[ref15] Guo J. Shi W. Li J. Zhai Z. (2021). Effects of poly-gamma-glutamic acid and poly-gamma-glutamic acid super absorbent polymer on the sandy loam soil hydro-physical properties. PLoS One 16:e0245365. doi: 10.1371/journal.pone.0245365 33434231 PMC7983855

[ref16] Guo J. Shi W. Wen L. Shi X. Li J. (2019). Effects of a super-absorbent polymer derived from poly-γ-glutamic acid on water infiltration, field water capacity, soil evaporation, and soil water-stable aggregates. Arch. Agron. Soil Sci. 66, 1627–1638. doi: 10.1080/03650340.2019.1686137

[ref17] Guo Z. Yang N. Zhu C. Gan L. (2017). Exogenously applied poly-gamma-glutamic acid alleviates salt stress in wheat seedlings by modulating ion balance and the antioxidant system. Environ. Sci. Pollut. Res. Int. 24, 6592–6598. doi: 10.1007/s11356-016-8295-4 28078521

[ref18] Hu L. Robert C. A. M. Cadot S. Zhang X. Ye M. Li B.. (2018). Root exudate metabolites drive plant-soil feedbacks on growth and defense by shaping the rhizosphere microbiota. Nat. Commun. 9:2738. doi: 10.1038/s41467-018-05122-7 30013066 PMC6048113

[ref19] Hu H. Wu C. Ge F. Ren Y. Li W. Li J. (2023). Poly-γ-glutamic acid-producing *Bacillus velezensis* fermentation can improve the feed properties of soybean meal. Food Biosci. 53:102503. doi: 10.1016/j.fbio.2023.102503

[ref20] Jaeger C. H. Lindow S. E. Miller W. Clark E. Firestone M. K. (1999). Mapping of sugar and amino acid availability in soil around roots with bacterial sensors of sucrose and tryptophan. Appl. Environ. Microbiol. 65, 2685–2690. doi: 10.1128/aem.65.6.2685-2690.1999 10347061 PMC91396

[ref21] Krishnamoorthy R. Kim K. Subramanian P. Senthilkumar M. Anandham R. Sa T. (2016). Arbuscular mycorrhizal fungi and associated bacteria isolated from salt-affected soil enhances the tolerance of maize to salinity in coastal reclamation soil. Agric. Ecosyst. Environ. 231, 233–239. doi: 10.1016/j.agee.2016.05.037

[ref22] Lagomarsino A. Mench M. Marabottini R. Pignataro A. Grego S. Renella G.. (2011). Copper distribution and hydrolase activities in a contaminated soil amended with dolomitic limestone and compost. Ecotoxicol. Environ. Saf. 74, 2013–2019. doi: 10.1016/j.ecoenv.2011.06.013 21798598

[ref23] Langmead B. Salzberg S. L. (2012). Fast gapped-read alignment with bowtie 2. Nat. Methods 9, 357–359. doi: 10.1038/nmeth.1923 22388286 PMC3322381

[ref24] Li C. Liao H. Xu L. Wang C. He N. Wang J.. (2022). The adjustment of life history strategies drives the ecological adaptations of soil microbiota to aridity. Mol. Ecol. 31, 2920–2934. doi: 10.1111/mec.16445 35344623

[ref25] Li R. Pang Z. Zhou Y. Fallah N. Hu C. Lin W.. (2020). Metagenomic analysis exploring taxonomic and functional diversity of soil microbial communities in sugarcane fields applied with organic fertilizer. Biomed. Res. Int. 2020, 1–11. doi: 10.1155/2020/9381506 PMC7596465 33145361

[ref26] Liang J. Shi W. He Z. Pang L. Zhang Y. (2019). Effects of poly-γ-glutamic acid on water use efficiency, cotton yield, and fiber quality in the sandy soil of southern Xinjiang, China. Agric. Water Manag. 218, 48–59. doi: 10.1016/j.agwat.2019.03.009

[ref27] Liu Y. Gong Y. Wang X. Hu Y. (2013). Volume fractal dimension of soil particles and relationships with soil physical-chemical properties and plant species diversity in an alpine grassland under different disturbance degrees. J. Arid. Land 5, 480–487. doi: 10.1007/s40333-013-0184-9

[ref28] Lu K. (1999). Analytical methods of soil and agricultural chemistry. Beijing: China Agricutural Science and Technology Press.

[ref29] Luo Z. Guo Y. Liu J. Qiu H. Zhao M. Zou W.. (2016). Microbial synthesis of poly-γ-glutamic acid: current progress, challenges, and future perspectives. Biotechnol. Biofuels 9:134. doi: 10.1186/s13068-016-0537-7 27366207 PMC4928254

[ref30] Ma H. Li P. Xiao N. Xia T. (2022). Poly-γ-glutamic acid promoted maize root development by affecting auxin signaling pathway and the abundance and diversity of rhizosphere microbial community. BMC Plant Biol. 22:521. doi: 10.1186/s12870-022-03908-y 36352394 PMC9647955

[ref31] Mansoor S. Khan T. Farooq I. Shah L. R. Sharma V. Sonne C.. (2022). Drought and global hunger: biotechnological interventions in sustainability and management. Planta 256:97. doi: 10.1007/s00425-022-04006-x 36219256

[ref32] Masuda Y. Yamanaka H. Xu Z. Shiratori Y. Aono T. Amachi S.. (2020). Diazotrophic *Anaeromyxobacter* isolates from soils. Appl. Environ. Microbiol. 86, e00956–e00920. doi: 10.1128/AEM.00956-20 32532868 PMC7414960

[ref33] Nock A. M. Wargo M. J. O'Toole G. A. (2016). Choline catabolism in *Burkholderia thailandensis* is regulated by multiple glutamine Amidotransferase 1-containing AraC family transcriptional regulators. J. Bacteriol. 198, 2503–2514. doi: 10.1128/jb.00372-16 27381916 PMC4999938

[ref34] Okutani F. Hamamoto S. Aoki Y. Nakayasu M. Nihei N. Nishimura T.. (2020). Rhizosphere modelling reveals spatiotemporal distribution of daidzein shaping soybean rhizosphere bacterial community. Plant Cell Environ. 43, 1036–1046. doi: 10.1111/pce.13708 31875335

[ref35] Qiao L. Wang X. Smith P. Fan J. Lu Y. Emmett B.. (2022). Soil quality both increases crop production and improves resilience to climate change. Nat. Clim. Chang. 12, 574–580. doi: 10.1038/s41558-022-01376-8

[ref36] Quan J. Zheng W. Tan J. Li Z. Wu M. Hong S.-B.. (2022). Glutamic acid and poly-γ-glutamic acid enhanced the heat resistance of Chinese cabbage (*Brassica rapa* L. ssp. pekinensis) by improving carotenoid biosynthesis, photosynthesis, and ROS signaling. Int. J. Mol. Sci. 23:11671. doi: 10.3390/ijms231911671 36232971 PMC9570168

[ref37] Raths R. Peta V. Bücking H. Baltrus D. A. (2019). Draft genome sequence of Massilia sp. strain MC02, isolated from a Sandy loam maize soil. Microbiol. Resour. Announc. 8, e00410–e00419. doi: 10.1128/mra.00410-19 31395629 PMC6687916

[ref38] Ren X. Yin S. Wang L. Tang J. J. S. O. T. T. E. (2022). Microplastics in plant-microbes-soil system: a review on recent studies. Sci. Total Environ. 816:151523. doi: 10.1016/j.scitotenv.2021.151523 34748830

[ref39] Song J. Brookes P. C. Shan S. Xu J. Liu X. J. G. (2022). Effects of remediation agents on microbial community structure and function in soil aggregates contaminated with heavy metals. Geoderma 425:116030. doi: 10.1016/j.geoderma.2022.116030

[ref40] Stephen Inbaraj B. Chiu C. P. Ho G. H. Yang J. Chen B. H. (2006). Removal of cationic dyes from aqueous solution using an anionic poly-γ-glutamic acid-based adsorbent. J. Hazard. Mater. 137, 226–234. doi: 10.1016/j.jhazmat.2006.01.057 16540239

[ref41] Su Y.-B. Peng B. Li H. Cheng Z.-X. Zhang T.-T. Zhu J.-X.. (2018). Pyruvate cycle increases aminoglycoside efficacy and provides respiratory energy in bacteria. Proc. Natl. Acad. Sci. USA 115, E1578–E1587. doi: 10.1073/pnas.1714645115 29382755 PMC5816162

[ref42] Swenson T. L. Jenkins S. Bowen B. P. Northen T. R. (2015). Untargeted soil metabolomics methods for analysis of extractable organic matter. Soil Biol. Biochem. 80, 189–198. doi: 10.1016/j.soilbio.2014.10.007

[ref43] Tahoun A. M. M. A. El-Enin M. M. A. Mancy A. G. Sheta M. H. Shaaban A. (2022). Integrative soil application of humic acid and foliar plant growth stimulants improves soil properties and wheat yield and quality in nutrient-poor Sandy soil of a semiarid region. J. Soil Sci. Plant Nutr. 22, 2857–2871. doi: 10.1007/s42729-022-00851-7 35528198 PMC9059912

[ref44] Tang J. (2011). Microbial Metabolomics. Curr. Genomics 12, 391–403. doi: 10.2174/138920211797248619 22379393 PMC3178908

[ref45] Turnbull A. L. Liu Y. Lazarovits G. (2012). Isolation of Bacteria from the rhizosphere and rhizoplane of potato (*Solanum tuberosum*) grown in two distinct soils using semi selective media and characterization of their biological properties. Am. J. Potato Res. 89, 294–305. doi: 10.1007/s12230-012-9253-4

[ref46] Upadhyay S. Raghubanshi A. S. (2020). “Determinants of soil carbon dynamics in urban ecosystems” in Urban Ecology, 299–314.

[ref47] Vinatzer B. A. Ofek M. Hadar Y. Minz D. (2012). Ecology of root colonizing Massilia (Oxalobacteraceae). PLoS One 7:e40117. doi: 10.1371/journal.pone.0040117 22808103 PMC3394795

[ref48] Wang Q. Chen S. Zhang J. Sun M. Liu Z. Yu Z. (2008). Co-producing lipopeptides and poly-γ-glutamic acid by solid-state fermentation of *Bacillus subtilis* using soybean and sweet potato residues and its biocontrol and fertilizer synergistic effects. Bioresour. Technol. 99, 3318–3323. doi: 10.1016/j.biortech.2007.05.052 17681465

[ref49] Wang X. Dong G. Liu X. Zhang S. Li C. Lu X.. (2020). Poly-gamma-glutamic acid-producing bacteria reduced cd uptake and effected the rhizosphere microbial communities of lettuce. J. Hazard. Mater. 398:123146. doi: 10.1016/j.jhazmat.2020.123146 32768845

[ref50] Wang Y. He Y. Zhan J. Li Z. (2022). Identification of soil particle size distribution in different sedimentary environments at river basin scale by fractal dimension. Sci. Rep. 12:10960. doi: 10.1038/s41598-022-15141-6 35768469 PMC9243010

[ref51] Withers E. Hill P. W. Chadwick D. R. Jones D. L. (2020). Use of untargeted metabolomics for assessing soil quality and microbial function. Soil Biol. Biochem. 143:107758. doi: 10.1016/j.soilbio.2020.107758

[ref52] Xu Z. Lei P. Feng X. Xu X. Liang J. Chi B.. (2014). Calcium involved in the poly (γ-glutamic acid)-mediated promotion of Chinese cabbage nitrogen metabolism. Plant Physiol. Biochem. 80, 144–152. doi: 10.1016/j.plaphy.2014.03.036 24762787

[ref53] Xu Z. Lei P. Pang X. Li H. Feng X. Xu H. (2017). Exogenous application of poly-gamma-glutamic acid enhances stress defense in *Brassica napus* L. seedlings by inducing cross-talks between ca (2+), H_2_O2, brassinolide, and jasmonic acid in leaves. Plant Physiol. Biochem. 118, 460–470. doi: 10.1016/j.plaphy.2017.07.015 28743039

[ref54] Xu Z. Ma J. Lei P. Wang Q. Feng X. Xu H. (2020). Poly-gamma-glutamic acid induces system tolerance to drought stress by promoting abscisic acid accumulation in *Brassica napus* L. Sci. Rep. 10:252. doi: 10.1038/s41598-019-57190-4 31937837 PMC6959327

[ref55] Yasmin H. Bano A. Wilson N. Nosheen A. Naz R. Hassan M. N.. (2022). Drought-tolerant Pseudomonas sp. showed differential expression of stress-responsive genes and induced drought tolerance in *Arabidopsis thaliana*. Physiol. Plant. 174:e13497. doi: 10.1111/ppl.13497 34245030

[ref56] Yin A. Jia Y. Qiu T. Gao M. Cheng S. Wang X.. (2018). Poly-γ-glutamic acid improves the drought resistance of maize seedlings by adjusting the soil moisture and microbial community structure. Appl. Soil Ecol. 129, 128–135. doi: 10.1016/j.apsoil.2018.05.008

[ref57] Yu F.-M. Jayawardena R. S. Thongklang N. Lv M.-L. Zhu X.-T. Zhao Q. (2022). Morel production associated with soil nitrogen-fixing and nitrifying microorganisms. J. Fungi 8:299. doi: 10.3390/jof8030299 PMC8950353 35330300

[ref58] Zhang H. Sun X. Dai M. (2022). Improving crop drought resistance with plant growth regulators and rhizobacteria: mechanisms, applications, and perspectives. Plant Commun. 3:100228. doi: 10.1016/j.xplc.2021.100228 35059626 PMC8760038

[ref59] Zhang L. Yang X. Gao D. Wang L. Li J. Wei Z.. (2017). Effects of poly-gamma-glutamic acid (gamma-PGA) on plant growth and its distribution in a controlled plant-soil system. Sci. Rep. 7:6090. doi: 10.1038/s41598-017-06248-2 28729559 PMC5519684

